# Synergistic Effects of a Novel Combination of Natural Compounds Prevent H_2_O_2_-Induced Oxidative Stress in Red Blood Cells

**DOI:** 10.3390/ijms26031334

**Published:** 2025-02-05

**Authors:** Giuditta Benincasa, Paola Bontempo, Ugo Trama, Claudio Napoli

**Affiliations:** 1 Department of Advanced Medical and Surgical Sciences (DAMSS), University of Campania “Luigi Vanvitelli”, 80138 Naples, Italy; direzione.immunoematologia@unicampania.it; 2 Department of Precision Medicine, University of Campania Luigi Vanvitelli, Via L. De Crecchio 7, 80138 Naples, Italy; paola.bontempo@unicampania.it; 3 Regional Pharmaceutical Unit, Campania Region, 80143 Naples, Italy; ugo.trama@regione.campania.it

**Keywords:** red blood cells, hydrogen peroxide, oxidative stress, natural compounds

## Abstract

Novel strategies to prevent the “storage lesions” of red blood cells (RBCs) are needed to prevent the risk of adverse effects after blood transfusion. One option could be the supplementation of stored blood bags with natural compounds that may increase the basal load of antioxidant protection and the shelf life of RBCs. In this pilot study, we investigated for the first time potential synergistic effects of a triple combination of well-known anti-oxidant compounds curcumin (curc), vitamin E (vit E), and vitamin C (vit C). Briefly, we established an ex vivo model of H_2_O_2_-induced oxidative stress and measured the hemolysis ratio (HR) (%) and thiobarbituric acid reactive substances (TBARS) levels in RBCs with or without pre-exposure for 30 min with increasing concentrations of curc, vit E, and vit C and then exposed to 10 mM H_2_O_2._ for 60 min. Exposure of RBCs to a triple combination of curc, vit E, and vit C at the highest concentration (100 µM) completely prevented H_2_O_2_-induced hemolysis. Surprisingly, we found that pre-treatment of RBCs with curc 100 µM alone completely prevented hemolysis as compared to vit E and vit C alone or in combination at the same concentration. On the other hand, pre-treatment with the triple combination of curc, vit E, and vit C 100 µM was required to totally prevent lipid peroxidation, as compared to curc 100 µM alone, supporting their synergistic effects in preventing RBCs membrane peroxidation. Further experiments are ongoing to investigate the anti-aging effects of the triple combination of curc, vit E, and vit C on cold-stored bags.

## 1. Introduction

The transfusion of red blood cells (RBCs) is a lifesaving treatment for severe anemia, trauma, or major surgery [1,2]. RBC-related “storage lesions” represent morphological, functional, and metabolic changes that RBCs progressively undergo upon collection, processing, and refrigerated storage in blood banks for clinical use [3,4,5]. Often, adverse events are caused by the transfusion of aged RBCs that are closer to their expiration date (42 days), wherein the effects of RBC storage lesions can contribute to poorer outcomes as compared to outcomes using fresher RBCs [6]. In order to address this unsolved clinical need, novel options to prevent storage lesions and extend the shelf life of RBCs are required. 

One approach to prevent storage lesions could be the supplementation of stored blood bags with natural compounds that may increase the basal load of antioxidant protection and shelf life of RBCs. Well-known anti-oxidant natural compounds, including vitamins [7,8,9], curcumin [7], quercetin [10,11], L-carnitine [12], açaì extract [13], and anthocyanin-rich extract (*Callistemon citrinus*) [14], have been widely investigated in accelerated aging models on human RBCs. However, there is a paucity of data about possible synergistic effects of specific combinations of natural compounds that may prevent oxidative stress better than one compound alone. 

Here, we evaluated for the first time the possible synergistic effects of a triple combination of well-known anti-oxidant natural compounds including curcumin (curc), L-ascorbic acid (vit C), and alpha-tocopherol (vit E) entering cells via related transmembrane porters. To this aim, we used an accelerated aging model of RBCs based on H_2_O_2_-induced oxidative stress and measured two markers of aging, the hemolysis ratio (HR) (%) and thiobarbituric acid reactive substances (TBARS) levels, which are basic biological toxicity tests widely used to evaluate the activity of natural compounds because they are rapid, reproducible, and less expensive than other tests, such as cell culturing. Due to the lack of methodological standardization in the literature, we also offer an original framework useful for easily establishing an H_2_O_2_-induced oxidative stress model in human RBCs. 

## 2. Results

### 2.1. Optimal Concentration of H_2_O_2_-Induced Oxidative Stress 

**Figure 1:**
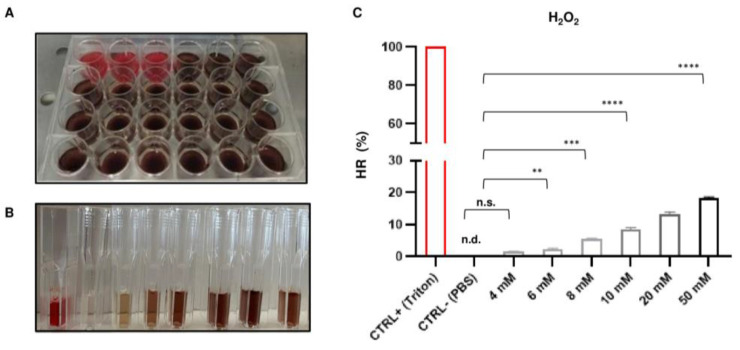
Selection of the H_2_O_2_ hemolysing dose. (**A**) A representative original image of a plate containing suspensions of RBCs without (red wells) or with (dark brown) H_2_O_2_. (**B**) A representative original image of cuvettes containing supernatants that were used to measure absorbance (**A**) at 540 nm after exposure to increasing concentrations of H_2_O_2_. From right to left in the image, contents of these cuvettes correspond to the bar graph in (**C**). (**C**) A bar graph showing the hemolysis ratios (HRs) (%) of RBCs obtained at increasing concentrations of H_2_O_2_. Values are the mean of independent experiments using RBCs from *n* = 3 blood donors assayed in triplicate (repeated-measures one-way ANOVA). ** *p* < 0.01; *** *p* < 0.001; **** *p* < 0.0001; n.d., not detectable; n.s., not significant.

In order to establish the optimal conditions of H_2_O_2_-induced oxidative stress, hemolysis assays were conducted upon exposure of RBCs to six increasing concentrations of peroxide. Data showed that hemolysis occurred in a concentration-dependent manner. In detail, we show a representative original plate in which we could observe the color change from red to dark brown in each well after incubation with increasing concentrations of H_2_O_2_ (from 4 to 50 mM) (Figure 1A). Moreover, a representative original picture shows the colour of supernatants that were collected after centrifuge that progressively changed from red towards dark brown in a direct proportional manner to increasing H_2_O_2_ concentration (Figure 1B). Except for 4 mM H_2_O_2_, RBC suspensions exposed to 6, 8, 10, 20, and 50 mM H_2_O_2_ had significantly increased HRs (%) as compared to that of the negative control (PBS) (*p* < 0.05) (Figure 1C). For the next experiments, we chose 10 mM H_2_O_2_ as a hemolysis-inducing low dose of peroxide to test the synergistic effects of natural compounds.

### 2.2. Evaluation of Synergistic Anti-Oxidant Effects of Curc, Vit E, and Vit C 

**Figure 2:**
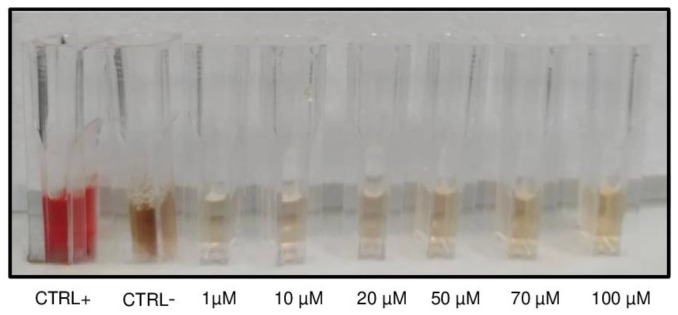
Preliminary evaluation of potential hemolytic effects. A representative original image of cuvettes containing supernatants from RBCs exposed to increasing concentrations of a triple combination of curc, vit E, and vit C.

In order to exclude any possibility of hemolysis upon exposure to the triple combination of natural compounds, we preliminarily evaluated the response of RBCs towards incubation with curc, vit E, and vit C ranging from 1 to 100 µM. As shown in Figure 2, we did not observe any sign of hemolysis as proven by the fact that the colour of supernatants in the cuvettes remained light yellow (no oxidative stress) after 1 h and 30 min of incubation at 37 °C in both lower (1–20 µM) and higher (50–70 µM) concentrations. 

**Figure 3:**
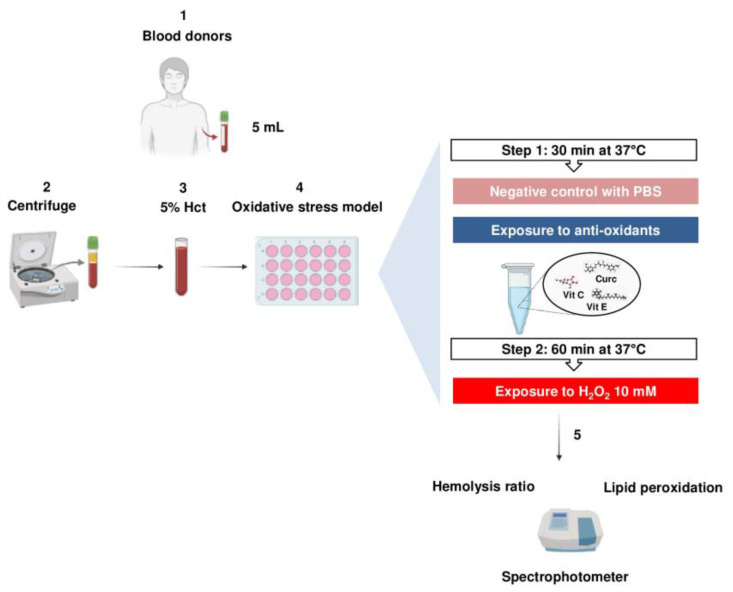
Experimental flowchart. Major details are provided in the Section 4.

After it was proven that the triple combination of curc, vit E, and vit C did not provoke hemolysis, we evaluated its ability to counteract the oxidative stress induced by peroxide. Therefore, we incubated RBCs with a triple combination of curc, vit E, and vit C at increasing concentrations (1–100 µM) for 30 min, and successively co-incubated 10 mM of H_2_O_2_ for 60 min. As the final step, we measured the HRs (%) of RBCs. The experimental workflow is shown in Figure 3. 

**Figure 4:**
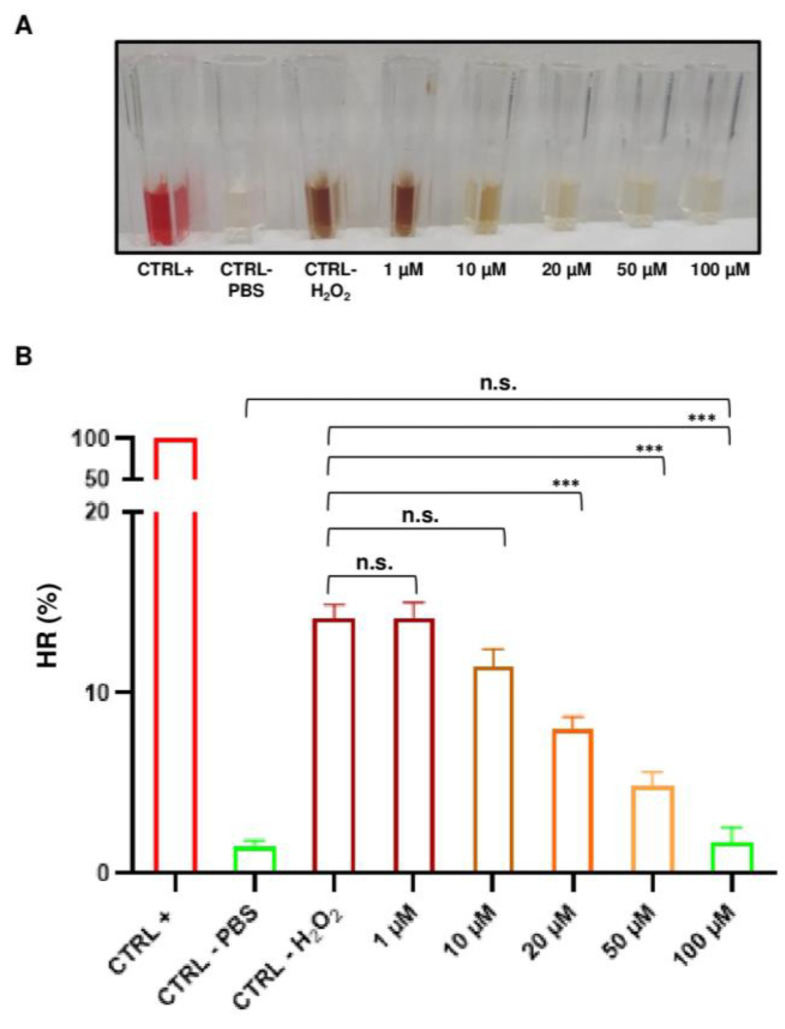
Synergistic anti-hemolytic effects of the triple combination of natural compounds at increasing concentrations. (**A**) A representative original image of cuvettes containing supernatants that were used to measure absorbance (**A**) at 540 nm after exposure to increasing concentrations of H_2_O_2_. (**B**) A bar graph showing relative hemolysis ratios (HRs) (%). Values are the mean of independent experiments using blood from *n* = 3 donors assayed in triplicate (repeated-measures 1-way ANOVA). *** *p* < 0.001; n.s., not significant.

In our experimental conditions, we observed that the HR (%) significantly decreased in a concentration-dependent manner. The ability to prevent hemolysis is observable in Figure 4A, which shows the colour of supernatants in cuvettes that progressively changed from dark brown (proven oxidative stress) towards light yellow (no oxidative stress). In detail, the triple combination of curc, vit E, and vit C at 20 µM (1-way ANOVA, *p* < 0.001), 50 µM (1-way ANOVA, *p* < 0.001), 70 µM (1-way ANOVA, *p* < 0.001), and 100 µM (1-way ANOVA, *p* < 0.001) significantly decreased the HR (%) of RBCs as compared to the H_2_O_2_-negative control (Figure 4B), whereas the triple combination of curc, vit E, and vit C at 1 µM and 10 µM did not prevent hemolysis as compared to the H_2_O_2_-negative control (1-way ANOVA, *p* > 0.05). 

Interestingly, we noted that the highest concentration of triple combination solution (100 µM) completely prevented oxidative stress as demonstrated by the fact that there was no significant difference between the HR (%) of RBCs pre-treated with the triple solution and that of the PBS negative control (1-way ANOVA, *p* > 0.05). Taken together, these data showed that starting from a concentration of 20 µM, a solution of curc, vit E, and vit C can prevent oxidative stress on RBCs in a dose-dependent manner, with total prevention reached at the highest concentration tested (100 µM). 

Starting from previous evidence on the use of higher concentrations of vitamins to evaluate their anti-aging effects in a model of cold-stored blood bags [8,9], we did not further consider 20 µM and 50 µM concentrations, but performed the following experiments using only pre-treatments at the highest concentration (100 µM). This choice is explained by our interest in evaluating the anti-aging effects of triple combinations of curc, vit E, and vit C using a model of cold-stored blood bags in future experimental workflows.

**Figure 5:**
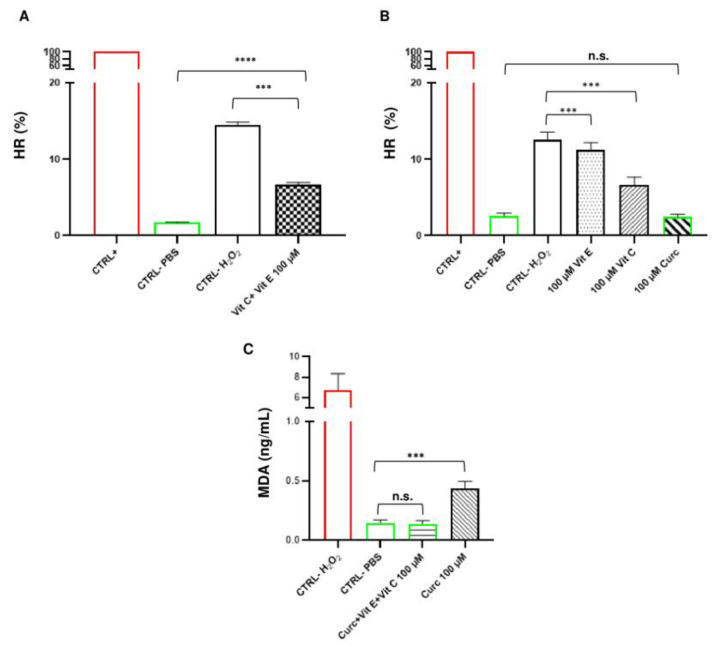
Evaluation of hemolysis and lipid peroxidation at the highest concentration of natural compounds. Bar graphs (**A**,**B**) show the hemolysis ratios (HRs) (%) of RBCs. Values are the mean of independent experiments using blood from *n* = 3 donors assayed in triplicate (repeated-measures 1-way ANOVA). (**C**) A bar graph showing MDA levels (ng/mL) obtained by pre-treatment with curc, vit E, and vit C in combination as compared to that of curc alone. Values are the mean of independent experiments using blood from *n* = 3 donors assayed in duplicate (repeated-measures 1-way ANOVA). *** *p* < 0.001; **** *p* < 0.0001. n.s., not significant.

Therefore, we measured the anti-hemolytic effects of RBCs pre-treatments with a double combination of vit C and vit E (100 µM) or single natural compounds of vit C or vit E (100 µM). We observed that pre-treatment with a combination of vit C and vit E significantly reduced the HR (%) compared to the negative control (1-way ANOVA, *p* < 0.0001), but did not totally prevent hemolysis (Figure 5A). Interestingly, pre-treatment of RBCs with curc alone was able to completely prevent hemolysis as compared to vit C and vit E alone (Figure 5B). This was proven by the fact that there was no significant difference between the HR (%) of RBCs pre-treated with curc 100 µM and that of the PBS negative control (1-way ANOVA, *p* > 0.05). These data support previous evidence that curc pre-treatment may be useful in maintaining the integrity of the RBC membrane [7]. 

Next, we chose to evaluate the triple combination of natural compounds versus curc alone in preventing lipid peroxidation of RBCs. In contrast to hemolysis, pre-treatment with curc 100 µM alone did not completely prevent lipid peroxidation. Indeed, MDA levels were statistically higher than those of the PBS negative control (1-way ANOVA, *p* < 0.001) (Figure 5C). Interestingly, the triple combination completely prevented lipid peroxidation, as shown by the fact that MDA levels were statistically similar to that of the negative control (PBS) (1-way ANOVA, *p* > 0.05) (Figure 5C). These data support that curc, vit E, and vit C may cooperate to prevent lipid peroxidation of RBC membranes with beneficial effects that are superior to those of curcumin alone. 

## 3. Discussion

Storage lesions are heterogeneous metabolic changes that occur in cold-stored blood bags leading to unavoidable aging-induced RBC membrane loss. In turn, loss of RBC integrity results in hemolysis and the formation of microparticles, which may contribute to complications associated with transfusion. As blood transfusion is one of the most common life-saving medical therapies, it is of paramount importance to better understand storage lesion-associated mechanisms and identify novel anti-aging treatments that may advance the current paradigm of transfusion medicine. In this context, we designed a preliminary study in order to evaluate for the first time the anti-oxidant effects of a triple combination of well-known anti-oxidant natural compounds, curc, vit E, and vit C, on RBCs by measuring hemolysis and lipid peroxidation as the main biomarkers of RBC-related membrane damages. Indeed, this pilot study was based on an accelerated model of aging and is part of our ongoing experiments directly on cold-stored blood bags.

The major findings of the present pilot study are as follows. (1) The exposure of RBCs to the triple combination of curc, vit E, and vit C at the highest concentration tested (100 µM) completely prevented hemolysis induced by H_2_O_2_ treatment. (2) The exposure of RBCs to curc alone accounted for the highest anti-hemolytic effects as compared to pre-treatment with a combination of vit C and vit E or pre-treatment with a single natural compound. (3) The exposure of RBCs to the triple combination of curc, vit E, and vit C at the highest concentration tested (100 µM) completely prevented lipid peroxidation as compared to the exposure of RBCs to curc alone. Taken together, these data support the possible synergistic effects of curc, vit E, and vit C in preventing storage lesions in RBCs. Our focus on the highest concentration of the triple combination of natural compounds arose from our ongoing experiments evaluating its possible anti-aging effects in a model of cold-stored blood bags (about 450 mL), and therefore required higher concentrations of natural compounds. The use of higher concentrations of natural compounds in a long-term experimental model is also supported by previous studies highlighting that vit C and an analogue of vitamin E (Trolox) had lasting anti-oxidant effects starting from a concentration of 125 µM (until 3125 μM) [8,9]. In detail, previous long-term studies showed that exposure to Trolox, a water-soluble analogue of vit E, significantly prevented hemolysis and lipid peroxidation in RBCs alone [8] or in combination with vit C [9]. However, a complete prevention of oxidative stress was not observed at the tested concentrations. Moreover, pre-treatment of RBCs with curc mitigated oxidative injury, membrane deformability, and elasticity better than vit C alone did [7]. 

Although many studies have evaluated the antioxidant effects of vitamins [7,8,9], curcumin [7], quercetin [10,11], L-carnitine [12], açaì extract [13], and *Callistemon citrinus* [14] for preventing storage lesions in RBCs or additional detrimental insults to human health [15,16,17], there is a paucity of data about the effects of such natural compounds in specific combinations that may yield synergistic anti-oxidant effects. This is the first ex vivo study evaluating the synergistic effects of a triple combination of curc, vit E, and vit C in preventing oxidative stress in RBCs using a range of both lower and higher concentrations. In this pilot study, we were surprised that the exposure of RBCs to curcumin alone had the ability to completely prevent hemolysis in the same manner the triple combination did, and that it had a higher anti-hemolytic effect as compared to vit C and vit E, alone or in combination. Curcumin, a yellow pigment commonly used as a spice and food coloring, is widely used as a nutraceutical compound owing to its anti-inflammatory properties [18]. One explanation could be that curcumin is effective in protecting RBCs from oxidative stress events at the level of cell membrane transport. Specifically, band 3 protein, which consists of a membrane domain-mediating anion exchange and a cytoplasmic domain, mainly contributes to protein–protein interactions by coupling the lipid bilayer to the underlying cytoskeleton through cysteine -SH groups [19]. On the other hand, the exposure of RBCs to curcumin alone was not able to completely prevent lipid peroxidation compared to exposure to the triple combination of natural compounds supporting synergistic effects among them. Vitamins C and E are both naturally occurring free radical scavengers. It is well known that vit C can protect membranes against peroxidative damage and enhances the effects of vit E by reducing tocopheroxyl radicals in human RBCs [8]. 

Although this study was not aimed at identifying the underlying mechanisms of this synergy, we hypothesize that the simultaneous presence of curc, vit E, and vit C amplifies the antioxidant effects of each individual compound forming a synergistic barrier for the lipid bilayer via neutralizing free radicals through the donation of electrons or hydrogen atoms and iron (II) chelating ability. Natural compounds act via complex molecular networks regulating cell senescence, inflammation, and the structural integrity of the membrane, which are key contributors to aging [7,18,20]. Among the potential molecular players regulating synergistic circuits in blood bags, we may hypothesize a role for the nuclear erythroid-2-related factor (NRF2), which is a key transcription factor during erythroid development affecting the expression of several antioxidant proteins, delaying cell senescence and preventing age-related diseases [21]. Additionally, the nuclear factor kappa-light-chain-enhancer of activated B cells (NF-kB) transcription factor is vital for promoting the survival of RBCs by delaying their apoptosis [22]. It is evident that more efforts should be made to evaluate which specific mechanisms of action can have anti-aging effects on RBCs in long-term models of storage lesions. 

Although this was an explorative study of small size, our results warrant further evaluation of the antioxidant effects of the triple combination of natural compounds directly in bags stored in blood biobanks [8,9]. Moreover, we provide an easy standardized methodological workflow aimed at helping researchers explore these novel beneficial anti-oxidants’ effects on RBCs or other hemoderivatives using a wide range of natural compounds.

## 4. Materials and Methods

### 4.1. Study Population 

As previously described [1,23,24,25,26], we enrolled periodic blood donors including non-smoker young males (30–35 years) at the Clinical Immunology, Immunohematology and Transfusion Medicine department of the University of Campania, “Luigi Vanvitelli” (Naples, Italy). RBCs were drawn from donor volunteers in accordance with guidelines from the Italian National Blood Centre (Blood Transfusion Service for donated blood).

### 4.2. Isolation of RBCs 

Peripheral blood (5 mL) was collected in heparinised tubes and used immediately. It was centrifuged at 1500 g for 10 min at 4 °C in a clinical centrifuge, and then both the supernatant and buffy coat were gently removed. The erythrocyte pellet was washed two times with phosphate-buffered saline (PBS) (0.9% NaCl in 10 mM sodium phosphate buffer, pH 7.4). After the second wash, packed erythrocytes were gently resuspended with PBS to give a 5% hematocrit. Importantly, suspensions of RBCs were pre-incubated at 37 °C for 10 min in the presence of 1 mM of sodium azide (NaN_3_) to inhibit catalase activity. This was a crucial step in establishing the oxidative stress model, as proven by the change in colour of RBCs from red to dark brown (Figure 1A), as clearly reported in previous protocols [27]. 

### 4.3. Establishment of the H_2_O_2_-Induced Oxidative Stress Model 

#### 4.3.1. Reagents 

Curcumin (#HY-N0005), vitamin E (#HY-W020044), and vitamin C (HY-B0166) were purchased from MCE Med Chem Express (Princeton, NJ, USA) and 30% H_2_O_2_ was purchased from Sigma-Aldrich (St. Louis, MO, USA) (#BCCD8661).

#### 4.3.2. Selection of the Suitable H_2_O_2_ Concentration

RBCs and enzymes are susceptible to oxidative damage, resulting in the peroxidation of membrane lipids and the release of hemoglobin (hemolysis). We chose H_2_O_2_ as the water-soluble oxidant because it can quickly permeate the membrane and partition in the cytosol leading to lipid membrane and enzyme oxidation [7,28]. Hemolysis assays were conducted as previously reported [7]. First, each 5% hematocrit was divided into ten aliquots: nine aliquots (after pre-exposure to NaN_3_) were treated with increasing concentrations of H_2_O_2_ (4 mM, 6 mM, 8 mM, 10 mM, 20 mM, and 50 mM) and one aliquot (without pre-exposure to NaN_3_) was treated with PBS (normal control). After 60 min at 37 °C, RBCs were immersed for 60 s in an ice bath and then centrifuged at 1500 g for 5 min at 4 °C. Supernatants were saved and their absorbance (A) was recorded at 540 nm to determine the release of hemoglobin. The hemolysis percentage was calculated using the formula:

Hemolysis Ratio (HR) (%) = A of sample − A of normal control (PBS)/A of positive control (Triton 1X) × 100

#### 4.3.3. Pre-Exposure to Natural Compounds and H_2_O_2-_Induced Oxidative Stress

RBC suspensions (5% hematocrit in PBS, 300 µL) were divided into three groups: the normal group (treated with PBS), the treatment group (triple combination of curc, vit E, and vit C) and the negative control group (treated with an H_2_O_2_-related hemolysing dose of 10 mM). For both the normal and negative control groups, suspensions of RBCs were pre-incubated with 100 µL PBS and 6 µL DMSO. In the treatment group, suspensions of RBCs were pre-incubated with 100 µL of a solution containing the triple combination of curc, vit E, and vit C at increasing concentrations (1 µM, 10 µM, 20 µM, 50 µM, 70 µM and 100 µM as the final concentration). In a subsequent experimental set, suspensions of RBCs were pre-incubated with 100 µL of a solution containing a single natural compound or specific double combinations at selected concentrations. Plates were incubated at 37 °C for 30 min. Subsequently, 400 µL of PBS was added to the normal group and 400 µL H_2_O_2_ solutions (pH 7.4) were added to the treatment and negative control groups. RBCs were then incubated at 37 °C for 1 h. After incubation, cell pellets were collected, immersed for 60 s in an ice bath, and then centrifuged at 1500 g for 5 min at 4 °C. Supernatants were saved and their A was recorded at 540 nm to determine the HR (%), as previously described.

#### 4.3.4. Lipid Peroxidation

Thiobarbituric acid reactive substances (TBARS) known as malondialdehyde (MDA) were measured using the Malondialdehyde (MDA) Colorimetric Assay Kit (Cell Samples) Elabscience (Houston, TX, USA) (#E-BC-K028-M), according to the manufacturer’s instructions. MDA is a widely used marker of lipid peroxidation [29]. The absorbance of pink chromophore produced during the reaction of thiobarbituric acid with malondialdehyde was measured at 535 nm. 

#### 4.3.5. Statistical Analysis 

Statistical procedures were performed with SPSS (version 20). Analysis of the dependent variable (absorbance at 540 nm) was performed with a repeated-measures 1-way ANOVA with “treatment” as a within-subject factor. The normality distribution of residuals was determined with Kolmogorov-Smirnov and Shapiro-Wilk tests. Sphericity was tested with the Mauchly test. If sphericity was violated (Mauchly test < 0.05) the Greenhouse–Geisser correction was applied. The alpha was 0.05. Post-hoc comparisons were performed with dependent *t*-tests corrected with the Bonferroni procedure. 

## Data Availability

Data are contained within the article. Raw data and materials are available from the authors upon reasonable request.
